# What are the impact and the optimal design of a physical prehabilitation program in patients with esophagogastric cancer awaiting surgery? A systematic review

**DOI:** 10.1186/s13102-021-00260-w

**Published:** 2021-03-25

**Authors:** Elise Piraux, Gregory Reychler, Louise Maertens de Noordhout, Patrice Forget, Yannick Deswysen, Gilles Caty

**Affiliations:** 1 Pôle de Neuro Musculo Skeletal Lab, Institut de Recherche Expérimentale et Clinique, Neuro Musculo Skeletal Lab, Université catholique de Louvain, Avenue Mounier 53, bte B1.53.07, 1200 Brussels, Belgium; 2 Pôle de Pneumologie, ORL & Dermatologie, Institut de Recherche Expérimentale et Clinique, Université Catholique de Louvain, Brussels, Belgium; 3 Clinical Neuroscience, Institute of Neurosciences, Université Catholique de Louvain, Brussels, Belgium; 4 Haute Ecole Léonard de Vinci, Parnasse-ISEI, Brussels, Belgium; 5 Secteur de kinésithérapie, Cliniques universitaires Saint-Luc, Brussels, Belgium; 6 Service de Pneumologie, Cliniques universitaires Saint-Luc, Brussels, Belgium; 7 Department of Anaesthetics, Institute of Applied Health Sciences, Epidemiology Group, University of Aberdeen, NHS Grampian, Aberdeen, UK; 8 Upper Gastrointestinal Surgery Unit, Cliniques Universitaires Saint-Luc, Brussels, Belgium; 9 Service de médecine physique et réadaptation, Cliniques universitaires Saint-Luc, Brussels, Belgium

**Keywords:** Esophagogastric cancer, Exercise therapy, Preoperative, Prehabilitation, Surgery, Systematic review

## Abstract

**Background:**

Substantial postoperative complications occur after tumor resection for esophagogastric cancers. Physical prehabilitation programs aim to prepare patients for surgery by improving their functional status with the aim of reducing postoperative complications. This systematic review aims to summarize the effects of physical prehabilitation programs on exercise capacity, muscle strength, respiratory muscle function, postoperative outcomes, and health-related quality of life and to determine the optimal design of such a program to improve these outcomes in esophagogastric cancer patients undergoing tumor resection.

**Methods:**

A systematic literature review was conducted using PubMed, The Cochrane Library, Scopus, and PEDro databases to identify studies evaluating the effects of physical prehabilitation program on exercise capacity, muscle strength, respiratory muscle function, postoperative complications, length of hospital stay, mortality, and health-related quality of life in patients with esophagogastric cancer awaiting surgery. Data from all studies meeting the inclusion criteria were extracted. The quality of each selected study was determined using the Downs and Black checklist.

**Results:**

Seven studies with 645 participants were included. The preoperative exercise program consisted of respiratory training alone in three studies, a combination of aerobic and resistance training in two studies, and a combination of respiratory, aerobic, and resistance training in two studies. Training frequency ranged from three times a day to twice a week and each session lasted between 20 and 75 min. Four studies were of fair quality and three of good quality. Some studies reported improvements in maximal inspiratory pressure, inspiratory muscle endurance, postoperative (pulmonary) complications, and length of hospital stay in the preoperative exercise group compared to the control group.

**Conclusion:**

This systematic review reports the current evidence for physical prehabilitation programs in patients with esophagogastric cancer awaiting surgery. However, due to the limited number of randomized controlled trials, the significant heterogeneity of exercise programs, and the questionable quality of the studies, higher quality randomized controlled trials are needed.

**Trial registration:**

PROSPERO Registration Number: CRD42020176353.

**Supplementary Information:**

The online version contains supplementary material available at 10.1186/s13102-021-00260-w.

## Background

Surgical resection is the mainstay of the curative treatment of localized and locally advanced esophagogastric cancer, alone or in combination with neoadjuvant chemoradiotherapy or chemotherapy [1, 2]. This surgery is a complex surgical procedure associated with high morbidity and mortality [3]. The risk of developing postoperative complications (PCs) for the patient increases with poor preoperative functional status [4]. Neoadjuvant therapy, physical inactivity, malnutrition, and increasing age deleteriously affect the patient’s functional status; therefore, the risk of the patient developing PCs after major surgery is increased [5–7]. Hence, strategies to optimize functional status with the aim of improving postoperative outcomes are of considerable importance [8].

In recent years, the use of the pre-surgical period has been suggested to effectively improve the patient’s physical status [8]. Surgical prehabilitation is a process on the continuum of care that occurs before surgery. Its objective is to improve baseline functional status to help the patient cope with the surgical stress imposed by surgery with the aim of improving postoperative outcomes and accelerating recovery [9]. Literature reports two typical cancer prehabilitation approaches, namely a unimodal and a multimodal regimens [10]. Although current trend seems to prefer multimodal prehabilitation approach to prepare patients for the upcoming surgery, both approaches have shown effectiveness in reducing perioperative morbidity and length of hospital stay (LOS), and improving functional capacity and recovery in cardiac, abdominal, or pulmonary surgical populations [11–15]. Nevertheless, systematic reviews in this field have reported a large heterogeneity in the content of prehabilitation programs. In view of this large heterogeneity, a better understanding of the impact of each intervention and how they can be implemented are a significant question to improve effectiveness of prehabilitation programs.

Structured exercise is part of both prehabilitation approaches and has been demonstrated to be very pertinent across the cancer experience [16]. This intervention is currently the most commonly used intervention in the prehabilitation literature and may consist of aerobic, resistance, and/or respiratory interventions. Studies investigating the effects of preoperative physical training on physical fitness and postoperative outcomes in patients with esophagogastric cancer awaiting surgery have been published in recent years [17, 18]. Some studies showed promising findings, with the reduction of postoperative complications while others reported conflicting results. A previous systematic review aimed to summarize these findings [19]; however, their findings were not specific to the effects of exercise intervention because they included studies with unimodal and multimodal regimens. Therefore, it seems relevant to consider to what extent a preoperative exercise program can be integrated into the care management of patients with esophagogastric cancer. In addition, the optimal design of preoperative exercise program to improve physical fitness and postoperative outcomes needs to be determined for patients with esophagogastric cancer.

Consequently, this systematic review aimed to summarize the effects of a preoperative exercise program on exercise capacity, muscle strength, respiratory muscle function, postoperative outcomes, and health-related quality of life (HRQoL) and to determine the most relevant exercise program design to improve these outcomes in patients with esophagogastric cancer undergoing tumor resection.

## Methods

### Protocol

This systematic review was registered on the PROSPERO database (https://www.crd.york.ac.uk/PROSPERO/, registration number CRD42020176353), and is reported in accordance with the Preferred Reporting Items for Systematic Reviews and Meta-Analyses (PRISMA) Statement [20].

### Systematic literature search

A systematic literature search was performed on the PubMed, The Cochrane Library for clinical trials, EMBASE (via Scopus), and PEDro databases from inception to April 2020. The search strategy was performed with no language restrictions and built using the following key terms: ‘esophageal cancer’, ‘gastric cancer’, ‘prehabilitation’, ‘physical prehabilitation’, ‘preoperative exercise’, and ‘physical exercise’. Using these key terms, an exhaustive list of keywords was created to build the specific search strategy for each database. Boolean operators ‘AND’ and ‘OR’ were used to connect key terms to obtain more focused and productive results. The exact search strategy for each database is reported in Additional file 1. A manual search of further relevant studies was performed in the reference lists of the selected studies and related review.

### Eligibility criteria

Studies were eligible for this systematic review according to the following PICOS eligibility criteria:

- Participants: Studies that included adults, aged 18 and over; diagnosed with esophageal, gastroesophageal junction, or gastric cancers; and scheduled for tumor resection were included.
- Intervention: Trials that applied preoperative physical training composed of respiratory, aerobic, and/or resistance training were included. Studies performing psychological or nutritional intervention in addition to the physical intervention were excluded.
- Comparison: Studies that compared a preoperative exercise program with usual care without exercise program or with another exercise modality were included.
- Outcomes: Studies that reported outcomes on exercise capacity, muscle strength, respiratory muscle function, PCs (including postoperative pulmonary complications (PPCs)), LOS, mortality, and HRQoL were included.
- Study design: Comparative study designs were included.

### Study selection

Articles identified from the four databases were collected. After removal of duplicates, two independent investigators (EP and LM) screened the titles and abstracts of records to determine their relevance, then reviewed the full texts to remove ineligible articles. When a study met all of the inclusion criteria, it was considered for the data extraction process. A third independent investigator (GC) was asked to decide if no consensus was reached between the other two investigators.

### Data collection process

The two investigators (EP and LM) independently extracted data from the selected studies. Study characteristics (authors, date of publication, and study design), sample characteristics (number of participants, age, sex, body mass index, type and stage of cancer, and cancer-related treatment), intervention data (setting, length, type, duration and frequency per sessions, attendance at exercise sessions, and exercise-related adverse events), and results related to exercise capacity, muscle strength, respiratory muscle function, postoperative outcomes and HRQoL were collected.

### Risk of bias in individual studies

The quality of each selected study was assessed independently by two investigators (EP and LM) using the Downs and Black checklist [21]. A third independent assessor (GC) was consulted in cases of discrepancy. The Downs and Black checklist is composed of 27 questions covering areas reporting quality, external validity, internal validity (bias and confounding), and power, with a maximum score of 28. A score less than 14 is as ‘poor quality’, between 14 and 18 as ‘fair quality’, between 19 and 23 as ‘good quality’, and ≥ 24 as ‘excellent quality’.

## Results

### Study selection

**Fig. 1:**
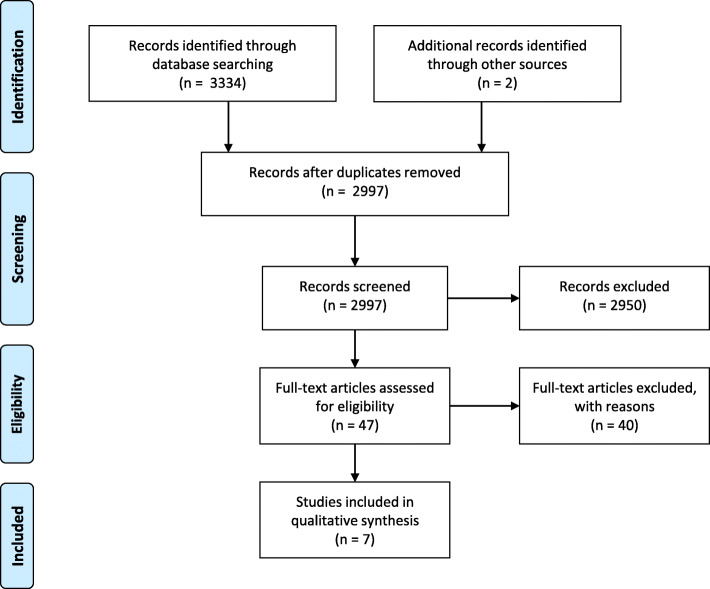
Literature search flow diagram

The PRISMA flow diagram is shown in Fig. 1. The search strategy yielded a total of 3334 citations from the four databases. Two additional studies were retrieved from manual searching within the reference lists. After removing the duplicates, 2997 citations were screened by reading title or abstract. The full texts of the 47 remaining relevant records were then analyzed for eligibility. Of these, 40 articles were removed because of not meeting the inclusion criteria. Seven studies were retained for inclusion [17, 18, 22–26].

### Study characteristics

The study characteristics are reported in Table 1. The seven eligible studies comprised three randomized controlled trials (RCTs) [18, 23, 24], two prospective non-randomized studies [17, 26], one retrospective study [22], and one prospective matching study [25]. Six of the seven studies compared an intervention group (IG) with a usual care control group (UC) receiving no preoperative exercise intervention [17, 18, 22, 23, 25, 26] and one study compared two IGs (IMT-high intensity group (IMT-HI) vs. IMT-endurance group (IMT-E)) [24]. Among the included studies, six were single-center, conducted in Japan [18, 22, 25], in the Netherlands [17, 24], and in Denmark [26]. One was a multicenter trial conducted in the Netherlands, Belgium, Ireland, and Finland [23].

**Table 1:** Study design and participant characteristics

Authors, year	Study design	Type and stage of cancer	Group	Sample (n)	Age (year)	Gender (M:F)	BMI (kg/m^2^)	Neoadjuvant treatment (n%)
Christensen et al., 2019 [26]	Non-randomized controlled trial	Gastroesophageal junction, I–III	IG UC	21 29	63.9 ± 8.2 65.5 ± 7.3	18:3 27:2	28.4 ± 5.6 27.8 ± 5.5	CT: 90%, CRT: 10% CT: 85%, CRT: 15%
Dettling et al., 2013 [17]	Pilot, non-randomized controlled study	Esophageal, NR	IG UC	44 39	65.1 ± 7.5 66.5 ± 9.6	33:11 29:10	24.9 ± 2.9 25.9 ± 2.9	CRT: 71% CRT: 44%
Inoue et al., 2013 [22]	Retrospective study	Esophageal, 0-IV	IG UC	63 37	67.4 ± 9.0 65.0 ± 7.8	53:10 34:3	20.7 ± 2.9 21.1 ± 2.7	CT: 68% CT: 43%
Valkenet et al., 2018 [23]	RCT	Esophageal, 0-IV	IG UC	120 121	63.7 ± 7.5 62.7 ± 8.9	89:31 97:24	26.7 ± 4.8 26.5 ± 5.2	CT: 8%, CRT: 78% CT: 10%, CRT: 78%
van Adrichem et al., 2014 [24]	Pilot, RCT	Esophageal, NR	IMT-HI IMT-E	20 19	62.7 ± 7.1 61.3 ± 7.3	15:5 14:5	23.9 (22.8–28.7) 25.7 (22.6–28.1)	CRT: 90% CRT: 95%
Yamana et al., 2015 [18]	RCT	Esophageal, 0-IV	IG UC	30 30	68.3 ± 7.6 65.9 ± 9.5	24:6 23:7	21.8 ± 2.7 20.9 ± 2.5	CT: 43%, RT: 10% CT: 50%, RT: 17%
Cho et al., 2014 [25]	Prospective matching study	Gastric, I-III	IG UC	18 54	63.1 (51–76) 66.1 (39–81)	18:0 51:3	26.7 (23.1–31.2) 25.6 (20.8–34.1)	None None

Age and BMI values are presented as mean ± SD or median (IQR). *BMI* body mass index, *CRT* chemoradiotherapy, *CT* chemotherapy, *F* female, *IG* intervention group, *IMT-HI/−E* inspiratory muscle training-high intensity/endurance group, *M* male, *NR* not reported, *RCT* randomized controlled trial, *RT* radiotherapy, *UC* usual care control group

### Participants

Participant characteristics are reported in Table 1. The included studies involved a total of 645 participants, of which 335 received preoperative intervention and 310 received usual care. Five studies included patients with esophageal cancer undergoing esophagectomy [17, 18, 22–24], one study reported on gastroesophageal junction cancer [26], and another study encompassed gastric patients [25]. All patients received neoadjuvant therapy in one study [26], the majority of patients in five studies [17, 18, 22–24], and none of the patients in one study [25]. Overall, significantly more men (81%) were included in the seven studies.

### Intervention characteristics

The characteristics of physical prehabilitation programs are summarized in Table 2. The prehabilitation interventions consisted of different exercise methods prior to surgery. Of these interventions, three studies focused only on a respiratory program during two or more weeks [17, 23, 24], two studies examined a combination of aerobic and resistance training for 11 to 19 weeks during the neoadjuvant chemotherapy [26] and for 4 weeks before surgery [25], and two studies combined aerobic, resistance, and respiratory training for at least 7 days [18, 22]. Four preoperative programs were conducted under supervision of qualified individuals in specialized centers [18, 22, 24, 26]. The remaining studies involved unsupervised and home sessions [23] or combined supervised and unsupervised sessions [17, 24]. Training frequency ranged from three times a day to twice a week and each session lasted between 20 and 75 min. Aerobic and resistance training was performed in four studies [18, 22, 25, 26] but only described in one study [26] in which subjects performed 21–28 min of high-intensity aerobic interval training [26]. Resistance training consisted of four exercises targeting major muscle groups and each was performed in three sets of 8 to 12 repetitions [26]. The respiratory component was performed in five studies and consisted only of IMT in three studies [17, 23, 24], general respiratory exercises without device in one study [18], or a combination of both in one study [22]. The intensity of IMT ranged from 30 to 80% of maximal inspiratory pressure (MIP) [17, 23, 24]. One study compared two IMT programs: the IMT-E consisted of IMT training at an initial intensity of 30% of MIP and the IMT-HI consisted of six cycles of six inspiratory maneuvers on an inspiratory threshold-loading device at 60–80% of MIP [24].

**Table 2:** Intervention characteristics and quality of studies

Authors, year	Group	Setting, supervision	Length of intervention	Frequency	Duration (min per session)	Exercise intervention	Attendance Adverse events	D&B
Christensen et al., 2019 [26]	IG	I, S	11–19 weeks	2x/week	75	Aerobic: HIIT on cycle ergometer: 4 × 4 min at 85–95% HRmax, 3 min active rest between, 21–28 min Resistance: Major muscle groups, 3 sets of 8–12 reps at 60–80% 1-RM	69% Worsening of the pre-exercise symptoms (fatigue, nausea, pain, dizziness) in 0.9–3.5% of all sessions	19
Dettling et al., 2013 [17]	IG	H, nS and I, S	25 ± 12 days^a^	7x/week	20	Respiratory: Threshold IMT device, 30% MIP increased by 10% if RPE < 5	NR 0	19
Inoue et al., 2013 [22]	IG	I, S	> 7 days	5x/week (aerobic-resistance) 3x /day (IMT)	40–60	Aerobic: 15 min on cycle ergometer Resistance: Lower limbs and abdominal muscles Respiratory: (1) IMT: 10 deep inspirations/set, 3 sets/time; (2) respiratory muscle stretching; (3) deep diaphragmatic breathing; (4) efficient coughing and huffing with abdominal contractions	NR NR	16
Valkenet et al., 2018 [23]	IG	H, nS	21 days^b^	2x/day	NR	Respiratory: Resistive inspiratory load device, 30 breaths at 60% MIP increased by 5% if RPE < 7	68% 0	19
van Adrichem et al., 2014 [24]	IMT-HI	I, S	3.7 weeks^b^	3x/week	N	Respiratory: 6 cycles of 6 inspiratory maneuvers on an inspiratory threshold-loading device at 60–80%, increased by 5% if RPE < 5	98% Tension headache	18
	IMT-E	H, nS and I, S	3.7 weeks^b^	7x/week	20	Respiratory: Threshold IMT device, 30% of MIP increased by 5% if RPE < 5	99/% Tension headache	
Yamana et al., 2015 [18]	IG	I, S	15 days^a^	5x/week	60	Aerobic: 20 min on cycle ergometer Resistance: Lower limbs and abdominal muscles Respiratory: (1) Respiratory muscle stretching; (2) deep diaphragmatic breathing; (3) efficient coughing and huffing with abdominal contractions	NR0	17
Cho et al., 2014 [25]	IG	NR	4 weeks	3–7x/week (aerobic) 1–2x/week (resistance)	NR	Aerobic: Treadmill, cycle ergometer, swimming, dancing, or jogging Resistance	NR 0	14

^a^mean ± SD, ^b^median; *1-RM* 1-repetition maximum, *D&B* downs and black checklist, *H* home-based, *HIIT* high-intensity interval training, *HRmax* maximum heart rate, *I* in-hospital, *IG* intervention group, *IMT* inspiratory muscle training, *IMT-HI/−E* inspiratory muscle training-high intensity/endurance group, *MIP* maximal inspiratory pressure (cmH2O), *NR* not reported, *nS* non-supervised, *RPE* rate of perceived exertion, *S* supervised

### Quality of studies

The quality assessment of the studies is shown in Table 2. Four studies were considered to be of fair quality [18, 22, 24, 25] and three of good quality [17, 23, 26]. All studies clearly reported objectives, described the outcomes to be measured in the introduction or methods section, and described the main findings. Two RCTs blinded evaluators [23, 24] but none of the studies blinded subjects because it was not feasible due to the intervention.

### Outcome measures

Exercise capacity and muscle strength were analyzed in one study by peak oxygen consumption and one-repetition maximum test, respectively [26]. Regarding respiratory muscle function, MIP was assessed in three studies with a hand-held respiratory pressure meter [17, 23, 24]. Inspiratory muscle endurance was assessed in two studies with an incremental threshold-loading device [17] and a hand-held device [23].

PCs, including PPCs, were evaluated in all included studies. Christensen et al. graded PCs according to the Clavien–Dindo classification and calculated the comprehensive complication index [26]. Dettling et al. reported the incidence of postoperative pneumonia, defined as a new infiltrative abnormality on chest X-ray accompanied by purulent sputum or fever in combination with the need for antibiotic treatment and incidence of other complications collected from a prospective database [17]. Inoue et al. classified PPCs, defined as in patients presenting at least four of the eight dichotomous factors defined by Reeve et al. [27], with the Clavien–Dindo classification [22]. Valkenet et al. measured the rate of postoperative pneumonia using the revised Uniform Pneumonia Score [23]. In the study by van Adrichem et al., PPCs were recorded using the criteria described by Kroenke et al. [28]. Yamana et al. classified PPCs according to the Clavien–Dindo classification and pneumonia using the Utrecht Pneumonia Scoring System. LOS and in-hospital mortality were reported in six [17, 22–26] and two [17, 23] of the seven studies, respectively.

HRQoL was assessed in two studies, one using the EuroQol-5D and the Short Form 12 questionnaires [23] and the other using the Functional Assessment of Cancer Therapy–Esophageal (FACT-E) questionnaire [26].

### Effectiveness of physical prehabilitation program

The results of each outcome extracted from the included studies are shown in Table 3.

**Table 3:** Results of the included studies

Authors, year	Exercise capacity, muscle strength, respiratory muscle function	Postoperative outcomes	HRQoL	
Christensen et al., 2019 [26]	In-group changes from baseline to post-prehabilitation ↗ Peak power (IG: + 12 watts) ↗ VO_2peak_ (IG: + 1.4 ml/min/kg) ↗ Leg press (IG: + 26.9 kg) ↗ Knee extension (IG: + 9.9 kg) ↗ Chest press (IG: + 5.1 kg) ↗ Seated row (IG: + 8.9 kg)	LOS (IG: 10 vs UC:9 days)^b^ PCs (CDC (grade ≥ 1), IG: 58% vs UC: 57%, RR 1.06, 95% CI 0.61–1.73) PCs (CCI score, IG: 20.9 vs UC: 20.9)^b^ Pneumonia (IG: 21% vs UC: 13%)	In-group changes from baseline to surgery ↗ EWB (IG: + 3.0) ↗ Esophageal cancer subscale (IG: + 8.8) ↗ FACT-E trial outcome index (IG: + 9.6) ↗ HRQoL total score (IG: + 12.6) Between-group changes from baseline to surgery ↔ HRQoL total score ↗ PWB (IG vs UC: mean difference 2.8)	
Dettling et al., 2013 [17]	In-group changes from baseline to post-prehabilitation ↗ MIP (IG: 74 to 91 cmH_2_0)^b^ ↗ P_m-peak_ (IG: 29 to 41 cmH_2_0)^b^ Between-groups comparison post-prehabilitation ↗ MIP (IG: 91 vs UC: 56 cmH_2_0)^b^ ↗ P_m-peak_ (IG: 41 vs UC: 25 cmH_2_0)^b^	↔ LOS (IG: 14 vs UC: 12 days)^b^ ↔ Pneumonia (IG: 25% vs UC: 23%) ↔ Other PCs ↔ In-hospital mortality (IG: 2% vs UC: 8%)	NA	
Inoue et al., 2013 [22]	NA	↔ LOS (IG: 41 vs UC: 50 days)^a^ ↘ PPCs (IG: 6% vs UC: 24%)	NA	
Valkenet et al., 2018 [23]	In-group changes from baseline to post-prehabilitation ↗ MIP (IG: 76 to 89 cmH_2_0 and UC: 74 to 80 cmH_2_0)^a^ ↗ P_i-end_ (IG: 4 min14 to 7 min19 and UC: 4 min20 to 5 min5)^a^ Between-groups changes from baseline to post-prehabilitation ↗ MIP (IG vs UC) ↗ P_i-end_ (IG vs UC)	↔ LOS (IG: 18 vs UC: 21 days)^a^ ↔ Pneumonia (IG: 39% vs UC: 36%) ↔ Other PPCs (IG: 35% vs UC: 33%) ↔ Other PCs (IG: 22% vs UC: 14%) ↔ In-hospital mortality (IG: 4% vs UC: 3%)	Between-groups changes from baseline to 4 weeks after surgery ↔ HRQoL (IG vs UC)	
van Adrichem et al., 2014 [24]	In-group changes from baseline to post-prehabilitation ↗ MIP (IMT-HI: 94 to 105 cmH_2_0 and IMT-E: 84 to 113 cmH_2_0)^b^ Between-groups ↔ MIP (IMT-HI vs IMT-E)	↘ LOS (IMT-HI: 14 vs IMT-E: 18 days)^b^ ↘ PPCs (IMT-HI: 20% vs IMT-E: 58%) ↔ Pneumonia (IG: 15% vs UC: 42%)	NA	
Yamana et al., 2015 [18]	NA	↘ PPCs (CDC (grade ≥ 1), IG: 27% vs UC 60%) ↘ Pneumonia (UPSS (Score ≥ 1) POD1, IG: 33% vs UC: 63%) ↔ Pneumonia (UPSS (Score ≥ 1) POD2, IG: 73% vs UC: 63%; POD3, IG: 73% vs UC: 50%; POD4, IG: 20% vs UC: 27%)	NA	
Cho et al., 2014 [25]	NA	↘ LOS (IG: 9 vs UC: 10 days)^b^ ↘ PCs (Intra-abdominal (all grades), IG: 6% vs UC: 33%) ↘ PCs (Intra-abdominal and wound infection (all grades), IG: 6% vs UC: 41%) ↔ PPCs (IG: 17% vs UC: 15%)	NA	

^a^mean, ^b^median; *CCI* comprehensive complication index, *CDC* Clavien-Dindo classification, *EWB* emotional well-being, *FACT–E* Functional Assessment of Cancer Therapy – Esophageal questionnaire, *HRQoL* health-related quality of life, *IG* intervention group, *IMT-HI/−E* inspiratory muscle training-high intensity/endurance group, *LOS* length of hospital stay, *MIP* maximal inspiratory pressure, *NA* not assessed, *P*_*i-end*_ inspiratory muscle endurance, *P*_*m-peak*_ maximal peak pressure, *PCs* postoperative complications, *PPCs* postoperative pulmonary complications, *POD1* postoperative day 1, *PWB* physical well-being, *RR* risk ratio, *UC* usual care control group, *UPSS* Utrecht pneumonia scoring system, *VO*_*2peak*_ peak oxygen consumption, vs versus

#### Effects on exercise capacity, muscle strength and, respiratory muscle function

Regarding exercise capacity and muscle strength, Christensen et al. reported significant improvements in peak oxygen consumption, peak power, and muscle strength (leg press, knee extension, chest press, and seated row) from baseline to post-prehabilitation in the IG [26].

MIP significantly increased in the IG compared to UC in two studies [17, 23]. In the third study, MIP increased significantly after interventions in IMT-HI and IMT-E groups but no significant difference was observed between groups [24]. Inspiratory muscle endurance differed significantly between the IG and UC groups in favor of the IG in two studies [17, 23].

#### Effects on postoperative outcomes

The incidence rate of pneumonia was lowered in the IG compared to UC at postoperative day 1 but not at postoperative days 2, 3 and 4 in one study [18]. No change in pneumonia incidence was observed in the three other studies [17, 23, 24]. Regarding other PPCs, two studies did not report a significant difference between groups [23, 25], whereas two others reported a significant reduction in the incidence rate of PPCs in the IG compared to UC [18, 22]. In addition, PPCs occurred significantly and about three times less after IMT-HI than IMT-E [24]. Concerning other PCs, one showed that a preoperative exercise program significantly reduced intra-abdominal and wound infection complications for all grades [25]. Another study did not reported a difference between groups [17].

Regarding LOS, four of the five studies comparing an IG to a CG showed no difference between groups [17, 22, 23, 26]. One demonstrated a significant difference between the IG and UC in favor of the IG [25]. In addition, in van Adrichem et al., patients in the IMT-HI group had a significantly shorter LOS than subjects in the IMT-E group [24].

In-hospital mortality was no different between the groups in the two studies reporting this outcome [17, 23].

#### Effects on quality of life

Valkenet et al. reported no significant difference in HRQoL between groups 4 weeks after surgery on the two scales used [23]. Conversely, Christensen et al. reported intra-group improvement in the IG from baseline to surgery for emotional well-being, esophageal cancer subscales, FACT-E trial outcome index score, and FACT-E total score. In addition, the evolution of physical well-being differed significantly between groups [26].

#### Attendance at exercise sessions and adverse events related to prehabilitation

Attendance at the exercise sessions, reported in three studies, ranged from 68 to 99% (Table 2) [23, 24, 26]. Regarding safety, four studies reported no exercise-related adverse event [17, 18, 23, 25]. In one study that performed an exercise program during neoadjuvant therapy, subjects reported worsening of pre-exercise symptoms (i.e., fatigue, nausea, pain, and dizziness) in 0.9 to 3.5% of all exercise sessions, with reported improvement in symptom burden in 3.8% to 14.2% of sessions after acute exercise [26]. However, these patient-reported symptoms do not appear to have interfered with exercise participation [26]. Van Adrichem et al. reported that among reasons for dropout, only tension headache was possibly related to the IMT [24].

## Discussion

This systematic review aimed to summarize the effects of physical prehabilitation programs (aerobic, resistance, and/or respiratory training) on exercise capacity, muscle strength, respiratory muscle function, postoperative outcomes, and HRQoL in patients with esophagogastric cancer awaiting surgery and to determine the most relevant preoperative exercise program design to improve these outcomes. Based on the results of the seven included studies, preoperative physical programs are feasible and safe and may be beneficial for improving exercise capacity, muscle strength, respiratory muscle function, HRQoL, PCs (including PPCs), and LOS in this population. However, these findings should be considered with caution due to the questionable methodological quality of the included studies (risk of bias due to internal validity, confounding factors, and external validity), which likely affected the results. In addition, the heterogeneity of the preoperative exercise program composition and the lack of information on the exercise program prescription prevented us from determining the optimal design of a preoperative exercise program. Since low exercise capacity and respiratory muscle weakness are associated with a higher risk of PCs [4, 29], improving preoperative exercise capacity and respiratory function with a physical prehabilitation program may be of interest in patients with esophagogastric cancer awaiting surgery. In the present systematic review, exercise capacity, muscle strength, and respiratory muscle function were assessed in few of the included studies, which precluded us from drawing relevant evidence.

Only Christensen et al. evaluated changes in exercise capacity and muscle strength after a preoperative aerobic and resistance training in patients with adenocarcinoma of the gastro-esophageal junction [26]. The IG showed significant improvements in peak oxygen consumption and muscle strength from baseline to post-prehabilitation. These findings are consistent with previous systematic reviews reporting improvement in physical capacity after a physical prehabilitation program in patients with gastrointestinal, non-small-cell lung, or various types of cancer [14, 30, 31]. However, the results of Christensen et al. should be interpreted with caution as they provided no information on the evolution of exercise capacity and muscle strength in the UC.

Regarding respiratory muscle function, two studies measuring this outcome reported a significant improvement in MIP and inspiratory muscle endurance after a preoperative IMT in favor of the IG [17, 23]. Findings from numerous preoperative IMT studies in patients awaiting thoracic and abdominal surgery have also shown improvement in MIP after an IMT [32, 33]. It was previously suggested that an improvement in respiratory muscle function achieved following an IMT led to a decrease in PPCs in patients undergoing cardiothoracic and upper abdominal surgery [33]. In the present systematic review, the positive influence of respiratory muscle function improvement on PPCs was not confirmed [16, 21].

One of the goals of a physical prehabilitation program is to reduce PCs, which are a major concern in upper abdominal surgeries. Given the conflicting observed results, no conclusion may be drawn on the effects of preoperative exercise training on reducing PCs. These discrepancies could be explained by the underpowered sample size that would limit the ability to detect significant effects and the lack of randomization in the majority of studies. Among the seven studies, one well-powered multicenter RCT reported no benefit in terms of PCs (including PPCs and pneumonia) in the IG compared to UC after an IMT [23]. These findings contrast those in literature in which pre-operative IMT resulted in a 50% reduction in PPCs in patients undergoing coronary bypass surgery [33]. However, esophagectomy is a highly invasive surgery that impacts the functioning of the diaphragm and could therefore explain that a relatively mild intervention such as IMT is insufficient to affect the postoperative outcome compared to other surgical populations such as cardiothoracic surgery [23]. The inclusion of aerobic and resistance training components to the preoperative exercise training could be more effective in reducing PCs by increasing cardiorespiratory capacity and muscle mass. The benefits of a preoperative combined prehabilitation program were demonstrated in patients with lung cancer undergoing tumor resection surgery [31]. Two included studies showed a significantly lower incidence rate of PPCs after a combined respiratory, aerobic, and resistance training in the IG compared to UC [18, 22]. However, the preoperative exercise programs of these studies did not provide complete details of the exercise intervention and their methodological quality is questionable. Therefore, these results should be interpreted with caution and more high-quality studies are expected to confirm their findings.

The reduction in LOS due to physical intervention was unclear after surgery in patients with esophagogastric cancer. Among the five studies that assessed this parameter, only a low quality study reported a significant reduction in LOS in favor of the IG over UC (9 vs. 10 days) [25]. In contrast, the well-powered RCT of Valkenet et al. found no statistically significant difference in LOS between groups [23]. In comparison, preoperative exercise has been found to shorten the LOS in patients with cancer undergoing lung surgery [14, 34] but not after intra-abdominal operations [11].

Patients with esophageal cancer have a poor preoperative quality of life due to treatment side effects, disease burden, and distress. These patients experience along-lasting deterioration in HRQoL after surgery [35]. Despite the effectiveness of exercise programs in improving HRQoL having been widely demonstrated during and after cancer treatment [36, 37], the evidence for the effect of preoperative exercise on improving HRQoL is weak and few studies investigated this outcome. Of the two studies evaluating HRQoL, one showed improvement in some domains of HRQoL, measured with FACT-E, from baseline to presurgery in the IG, and only change in physical well-being differed significantly between groups [26]. The other study found no difference between the groups 4 weeks after surgery [23]. This lack of evidence of HRQoL improvement after a physical prehabilitation program is consistent with the literature [31, 38].

Currently, no guidelines outline a consistent protocol for preoperative physical programs [39]. The heterogeneity encountered in this review prevented us from determining the optimal exercise program design in terms of frequency, intensity, time and type. However, some extracted data regarding duration and setting can be highlighted. Concerning the duration, all preoperative exercises were performed within one to four weeks prior to surgery with the exception of the study by Christensen et al., where patients trained during neoadjuvant treatment for 11 to 19 weeks [26]. This study demonstrated the feasibility and safety of a preoperative exercise program during neoadjuvant chemotherapy in patients with gastro-esophageal junction adenocarcinoma. Given these findings, it would be interesting to implement the physical prehabilitation program at the beginning of the neoadjuvant treatment and to continue it until surgery to counter the side effects of neoadjuvant therapy and then increase the patient’s capacity up to surgery. This has previously been reported by Singh et al., who included patients with localized rectal cancer awaiting surgery [40]. Nevertheless, other studies including a preoperative exercise program between one and four weeks displayed some interesting results. Regarding the setting, we observed the feasibility and safety of carrying out a preoperative exercise program either hospital-based, home-based, or a combination of both but there are too few good quality studies in the present field to highlight the advantages of any of the possibilities. However, among the studies reporting a significant decrease in the incidence of PPCs in favor of the IG [18, 22, 24], all were supervised and hospital-based, hypothesizing that this type of setting might be more effective than home-based program or a combination of both to reduce PPCs. However, transportation-related issues (finding/paying for parking, arranging transportation) are the greatest barrier reported to participating in a prehabilitation program [41], and implementing hospital-based exercise sessions could therefore negatively influence patient attendance at prehabilitation. Yet, patient attendance is essential for maximizing the effectiveness of the exercise training since success is related to the amount of exercise performed. In this study, attendance rates were only reported in three studies (69 and 98% after in-hospital exercise sessions, 99% after a combination of hospital and home sessions, and 68% after sessions at home), which prevented us from observing whether patient adherence to exercise sessions is better in the hospital or at home [23, 24, 26]. Tele-rehabilitation is an effective strategy to overcome these transportation-related barriers to exercise by offering a home exercise program combined with remote supervision through telecommunication services. This method of program delivery has been shown to be effective in various chronic conditions [42, 43] and, recently, a pilot study reported the feasibility, safety, and benefits of a preoperative tele-rehabilitation program in patients with esophagogastric cancer, but the authors concluded that more RCTs are needed [44].

This systematic review encountered various limitations. The main limitation of this systematic review was the heterogeneity of the exercise protocol in the included studies. In addition, there was a significant lack of information on the exercise program prescription, limiting interpretation of the results. Without these details, it is impossible to replicate and validate the intervention or to implement it in clinical practice. In the future, authors should report the specific details of the exercise program conducted in their study. Along this line, the optimal preoperative physical training (setting, type, length of intervention, and intensity) also remains to be established. Other causes of heterogeneity may include the difference or absence of neoadjuvant therapies and different types of surgeries, resulting in a possible different degree of postoperative outcomes. In addition, the definition of postoperative complications was different between studies because there is no standardized and comprehensive classification. Second, the quality of the studies varied between fair and good, for which more than half of the studies were not randomized, and the majority did not blind the outcomes assessor, nor were they adequately powered. In the future, higher quality RCTs with an appropriate sample size will be needed to evaluate the impact of a physical prehabilitation program in patients with esophagogastric cancer. Finally, this work focused only on unimodal studies performing a preoperative exercise program in order to determine, among other things, its specific effects; therefore, results cannot be generalized to multimodal approaches, even though the current trend seems to be towards a multimodal approach.

## Conclusions

This systematic review reported the current evidence for physical prehabilitation programs in patients with esophagogastric cancer awaiting surgery. Preoperative exercise programs are feasible and safe in this population. However, it was not possible to draw a definitive conclusion about either the effects of physical prehabilitation programs or the optimal exercise program design in patients with esophagogastric cancer undergoing surgery due to the limited number of RCTs, the significant heterogeneity of the exercise programs, and the questionable quality of the studies. Therefore, higher quality RCTs are needed to clarify the role of physical prehabilitation programs for these patients.

## Supplementary Information

**Additional file 1.** . Search Strategies used in the four databases

## Data Availability

All data generated or analyzed during this study are included in this published article.

## Abbreviations

1-RM: 1-repetition maximum
BMI: Body mass index
CCI: Comprehensive complication index
CDC: Clavien-Dindo classification
CRT: Chemoradiotherapy
CT: Chemotherapy
D&B: Downs and black checklist
EWB: Emotional well-being
F: Female
FACT-E: Functional Assessment of Cancer Therapy–Esophageal
H: Home-based
HIIT: High-intensity interval training
HRmax: Maximum heart rate
HRQoL: Health-related quality of life
I: In-hospital
IG: Intervention group
IMT: Inspiratory muscle training
IMT-HI/−E: Inspiratory muscle training-high intensity/endurance group
LOS: Length of hospital stay
NA: Not assessed
M: Male
MIP: Maximal inspiratory pressure
NR: Not reported
nS: Non-supervised
PICOS: Participants, interventions, comparisons, outcomes, study design
PCs: Pulmonary complications
Pi-end: Inspiratory muscle endurance
Pm-Peak: Maximal peak pressure
POD1: Postoperative day 1
PPCs: Postoperative pulmonary complications
PRISMA: Preferred reporting items for systematic reviews and meta-analyses
PROSPERO: International prospective register of systematic reviews
PWB: Physical well-being
RCTs: Randomized controlled trials
RPE: Rate of perceived exertion
RR: Risk ratio
RT: Radiotherapy
S: Supervised
UC: Usual care control group
UPSS: Utrecht pneumonia scoring system
VO2peak: Peak oxygen consumption

## References

[1] Ajani JA, D'Amico TA, Almhanna K, Bentrem DJ, Chao J, Das P (2016). Gastric cancer, version 3.2016, NCCN clinical practice guidelines in oncology. J Natl Compr Cancer Netw.

[2] Ajani JA, D'Amico TA, Bentrem DJ, Chao J, Corvera C, Das P (2019). Esophageal and Esophagogastric junction cancers, version 2.2019, NCCN clinical practice guidelines in oncology. J Natl Compr Cancer Netw.

[3] Low DE, Kuppusamy MK, Alderson D, Cecconello I, Chang AC, Darling G (2019). Benchmarking complications associated with Esophagectomy. Ann Surg.

[4] Patel N, Powell AG, Wheat JR, Brown C, Appadurai IR, Davies RG (2019). Cardiopulmonary fitness predicts postoperative major morbidity after esophagectomy for patients with cancer. Phys Rep.

[5] Jack S, West MA, Raw D, Marwood S, Ambler G, Cope TM (2014). The effect of neoadjuvant chemotherapy on physical fitness and survival in patients undergoing oesophagogastric cancer surgery. Eur J Surg Oncol.

[6] Milanovic Z, Pantelic S, Trajkovic N, Sporis G, Kostic R, James N (2013). Age-related decrease in physical activity and functional fitness among elderly men and women. Clin Interv Aging.

[7] Feeney C, Reynolds JV, Hussey J (2011). Preoperative physical activity levels and postoperative pulmonary complications post-esophagectomy. Dis Esophagus.

[8] Carli F, Zavorsky GS (2005). Optimizing functional exercise capacity in the elderly surgical population. Curr Opin Clin Nutr Metab Care.

[9] Carli F, Gillis C, Scheede-Bergdahl C (2017). Promoting a culture of prehabilitation for the surgical cancer patient. Acta Oncol.

[10] Lukez A, Baima J (2020). The role and scope of Prehabilitation in cancer care. Semin Oncol Nurs.

[11] Moran J, Guinan E, McCormick P, Larkin J, Mockler D, Hussey J (2016). The ability of prehabilitation to influence postoperative outcome after intra-abdominal operation: a systematic review and meta-analysis. Surgery..

[12] Hulzebos EH, Smit Y, Helders PP, van Meeteren NL (2012). Preoperative physical therapy for elective cardiac surgery patients. Cochrane Database Syst Rev.

[13] Hijazi Y, Gondal U, Aziz O (2017). A systematic review of prehabilitation programs in abdominal cancer surgery. Int J Surg.

[14] Rosero ID, Ramirez-Velez R, Lucia A, Martinez-Velilla N, Santos-Lozano A, Valenzuela PL, et al. Systematic review and meta-analysis of randomized, controlled trials on preoperative physical exercise interventions in patients with non-small-cell lung cancer. Cancers (Basel). 2019;11(7):944. 10.3390/cancers11070944. 10.3390/cancers11070944 PMC6678369 31284372

[15] Pouwels S, Fiddelaers J, Teijink JA, Woorst JF, Siebenga J, Smeenk FW (2015). Preoperative exercise therapy in lung surgery patients: a systematic review. Respir Med.

[16] Courneya KS, Friedenreich CM (2007). Physical activity and cancer control. Semin Oncol Nurs.

[17] Dettling DS, Van der Schaaf M, Blom RLGM, Nollet F, Busch ORC, Van Berge Henegouwen MI (2013). Feasibility and effectiveness of pre-operative inspiratory muscle training in patients undergoing Oesophagectomy: a pilot study. Physiother Res Int.

[18] Yamana I, Takeno S, Hashimoto T, Maki K, Shibata R, Shiwaku H (2015). Randomized controlled study to evaluate the efficacy of a preoperative respiratory rehabilitation program to prevent postoperative pulmonary complications after Esophagectomy. Dig Surg.

[19] Bolger JC, Loughney L, Tully R, Cunningham M, Keogh S, McCaffrey N, et al. Perioperative prehabilitation and rehabilitation in esophagogastric malignancies: a systematic review. Dis Esophagus. 2019;32(9). 10.1093/dote/doz058. 10.1093/dote/doz058 31206582

[20] Liberati A, Altman DG, Tetzlaff J, Mulrow C, Gøtzsche PC, Ioannidis JP (2009). The PRISMA statement for reporting systematic reviews and meta-analyses of studies that evaluate healthcare interventions: explanation and elaboration. Bmj..

[21] Downs SH, Black N (1998). The feasibility of creating a checklist for the assessment of the methodological quality both of randomised and non-randomised studies of health care interventions. J Epidemiol Community Health.

[22] Inoue J, Ono R, Makiura D, Kashiwa-Motoyama M, Miura Y, Usami M (2013). Prevention of postoperative pulmonary complications through intensive preoperative respiratory rehabilitation in patients with esophageal cancer. Dis Esophagus.

[23] Valkenet K, Trappenburg JCA, Ruurda JP, Guinan EM, Reynolds JV, Nafteux P (2018). Multicentre randomized clinical trial of inspiratory muscle training versus usual care before surgery for oesophageal cancer. Br J Surg.

[24] van Adrichem EJ, Meulenbroek RL, Plukker JTM, Groen H, Van Weert E (2014). Comparison of two preoperative inspiratory muscle training programs to prevent pulmonary complications in patients undergoing esophagectomy: a randomized controlled pilot study. Ann Surg Oncol.

[25] Cho H, Yoshikawa T, Oba MS, Hirabayashi N, Shirai J, Aoyama T (2014). Matched pair analysis to examine the effects of a planned preoperative exercise program in early gastric cancer patients with metabolic syndrome to reduce operative risk: the adjuvant exercise for general elective surgery (AEGES) study group. Ann Surg Oncol.

[26] Christensen JF, Simonsen C, Banck-Petersen A, Thorsen-Streit S, Herrstedt A, Djurhuus SS (2019). Safety and feasibility of preoperative exercise training during neoadjuvant treatment before surgery for adenocarcinoma of the gastro-oesophageal junction. BJS Open.

[27] Reeve JC, Nicol K, Stiller K, McPherson KM, Denehy L (2008). Does physiotherapy reduce the incidence of postoperative complications in patients following pulmonary resection via thoracotomy? A protocol for a randomised controlled trial. J Cardiothorac Surg.

[28] Kroenke K, Lawrence VA, Theroux JF, Tuley MR (1992). Operative risk in patients with severe obstructive pulmonary disease. Arch Intern Med.

[29] Nomori H, Kobayashi R, Fuyuno G, Morinaga S, Yashima H (1994). Preoperative respiratory muscle training: assessment in thoracic surgery patients with special reference to postoperative pulmonary complications. Chest..

[30] Lau CSM, Chamberlain RS. Prehabilitation programs improve exercise capacity before and after surgery in gastrointestinal cancer surgery patients: a meta-analysis. J Gastrointest Surg. 2019;24(12):2829–37. 10.1007/s11605-019-04436-1. 10.1007/s11605-019-04436-1 31768827

[31] Piraux E, Caty G, Reychler G (2018). Effects of preoperative combined aerobic and resistance exercise training in cancer patients undergoing tumour resection surgery: a systematic review of randomised trials. Surg Oncol.

[32] Kulkarni SR, Fletcher E, McConnell AK, Poskitt KR, Whyman MR (2010). Pre-operative inspiratory muscle training preserves postoperative inspiratory muscle strength following major abdominal surgery - a randomised pilot study. Ann R Coll Surg Engl.

[33] Hulzebos EH, Helders PJ, Favie NJ, De Bie RA, de la Brutel A, Van Meeteren NL (2006). Preoperative intensive inspiratory muscle training to prevent postoperative pulmonary complications in high-risk patients undergoing CABG surgery: a randomized clinical trial. Jama..

[34] Steffens D, Beckenkamp PR, Hancock M, Solomon M, Young J (2018). Preoperative exercise halves the postoperative complication rate in patients with lung cancer: a systematic review of the effect of exercise on complications, length of stay and quality of life in patients with cancer. Br J Sports Med.

[35] Jacobs M, Macefield RC, Elbers RG, Sitnikova K, Korfage IJ, Smets EM (2014). Meta-analysis shows clinically relevant and long-lasting deterioration in health-related quality of life after esophageal cancer surgery. Qual Life Res.

[36] Mishra SI, Scherer RW, Snyder C, Geigle PM, Berlanstein DR, Topaloglu O. Exercise interventions on health-related quality of life for people with cancer during active treatment. Cochrane Database Syst Rev. 2012;2012(8):Cd008465. 10.1002/14651858.CD008465.pub2. 10.1002/14651858.CD008465.pub2 PMC7389071 22895974

[37] Gerritsen JK, Vincent AJ (2016). Exercise improves quality of life in patients with cancer: a systematic review and meta-analysis of randomised controlled trials. Br J Sports Med.

[38] Vermillion SA, James A, Dorrell RD, Brubaker P, Mihalko SL, Hill AR (2018). Preoperative exercise therapy for gastrointestinal cancer patients: a systematic review. Syst Rev.

[39] Scheede-Bergdahl C, Minnella EM, Carli F (2019). Multi-modal prehabilitation: addressing the why, when, what, how, who and where next?. Anaesthesia..

[40] Singh F, Newton RU, Baker MK, Spry NA, Taaffe DR, Galvao DA (2017). Feasibility and efficacy of presurgical exercise in survivors of rectal cancer scheduled to receive curative resection. Clin Colorectal Cancer.

[41] Ferreira V, Agnihotram RV, Bergdahl A, van Rooijen SJ, Awasthi R, Carli F (2018). Maximizing patient adherence to prehabilitation: what do the patients say?. Support Care Cancer.

[42] Galiano-Castillo N, Cantarero-Villanueva I, Fernandez-Lao C, Ariza-Garcia A, Diaz-Rodriguez L, Del-Moral-Avila R (2016). Telehealth system: a randomized controlled trial evaluating the impact of an internet-based exercise intervention on quality of life, pain, muscle strength, and fatigue in breast cancer survivors. Cancer..

[43] van Egmond MA, van der Schaaf M, Vredeveld T, Vollenbroek-Hutten MMR, van Berge Henegouwen MI, Klinkenbijl JHG (2018). Effectiveness of physiotherapy with telerehabilitation in surgical patients: a systematic review and meta-analysis. Physiotherapy..

[44] Piraux E, Caty G, Reychler G, Forget P, Deswysen Y. Feasibility and preliminary effectiveness of a Tele-Prehabilitation program in Esophagogastric cancer patients. J Clin Med. 2020;9(7):2176. 10.3390/jcm9072176. 10.3390/jcm9072176 PMC7408844 32660126

