# Supplementary material for: What are the impact and the optimal design of a physical prehabilitation program in patients with esophagogastric cancer awaiting surgery? A systematic review
Source: BMC Sports Sci Med Rehabil. 2021 Mar 25;13:33. doi: 10.1186/s13102-021-00260-w (PMC7993458; doi:10.1186/s13102-021-00260-w)
Supplement: Supplementary file 1 — Additional file 1. . Search Strategies used in the four databases [file 13102_2021_260_MOESM1_ESM.docx]

## Additional file 1. Search Strategies used in the four databases

Search strategy Pubmed:

(esophag* OR oesophag* OR gastroesophag* OR upper gastrointestinal OR gastric OR esophagogastric) AND (cancer OR tumour OR neoplasms OR malignanc* OR adenocarcinoma) AND (Prehabilitation OR pre-rehabilitation OR pre-operative OR preoperative OR presurgical OR surgery OR pre-conditioning OR esophagectomy OR gastrectomy) AND (training OR physical exercise OR fitness OR strength training OR aerobic OR exercise therapy OR physical training OR strength exercise OR strengthening program OR breathing exercise OR inspiratory muscle training OR endurance training OR resistance training OR resistance exercise OR rehabilitation)

Search strategy the Cochrane Library for clinical trial:

(esophag* OR oesophag* OR gastroesophag* OR upper gastrointestinal OR gastric OR esophagogastric) AND (cancer OR tumour OR neoplasms OR malignanc* OR adenocarcinoma) AND (Prehabilitation OR pre-rehabilitation OR pre-operative OR preoperative OR presurgical OR surgery OR pre-conditioning OR esophagectomy OR gastrectomy) AND (training OR physical exercise OR fitness OR strength training OR aerobic OR exercise therapy OR physical training OR strength exercise OR strengthening program OR breathing exercise OR inspiratory muscle training OR endurance training OR resistance training OR resistance exercise OR rehabilitation)

Search strategy EMBASE (via Scopus):

( TITLE-ABS-KEY ( ( esophag* ) OR ( oesophag* ) OR ( gastroesophag* ) OR ( "upper gastrointestinal" ) ) AND TITLE-ABS-KEY ( ( cancer ) OR ( tumour ) OR ( neoplasms ) OR ( malignanc* ) OR ( adenocarcinoma ) ) AND TITLE-ABS-KEY ( ( prehabilitation ) OR ( pre-rehabilitation ) OR ( pre-operative ) OR ( preoperative ) OR ( presurgical ) OR ( surgery ) OR ( pre-conditioning ) OR ( esophagectomy ) ) AND TITLE-ABS-KEY ( ( training ) OR ( "physical exercise" ) OR ( fitness ) OR ( "strength training" ) OR ( aerobic ) OR ( "exercise therapy" ) OR ( "physical training" ) OR ( "strength exercise" ) ) AND TITLE-ABS-KEY ( ( "strengthening program" ) OR ( "breathing exercise" ) OR ( "inspiratory muscle training" ) OR ( "resistance training" ) OR ( endurance ) ) )

Search strategy PEDro:

Abstract and title: Prehabilitation

Subdiscipline: Oncology

Method: clinical trial
